# Development of a Functional Assessment of Chronic Illness Therapy item library and primary symptom list for the assessment of patient-reported adverse events associated with immune checkpoint modulators

**DOI:** 10.20517/2394-4722.2019.38

**Published:** 2020-03-13

**Authors:** Kimberly A. Webster, Mary L. O’Connor, Aaron R. Hansen, Sheetal Kircher, Heather S. L. Jim, Adam P. Dicker, Monika Janda, Kari Ala-leppilampi, Clifton O. Bingham, Josephine Feliciano, Norah Lynn Henry, Laurie E. Steffen McLouth, David Cella

**Affiliations:** 1 Department of Medical Social Sciences, Northwestern University Feinberg School of Medicine, Chicago, IL 60611, USA.; 2 Princess Margaret Cancer Center, University Health Network, Toronto M5G 1Z5, Canada.; 3 Robert H Lurie Comprehensive Cancer Center, Northwestern University, Chicago, IL 60611, USA.; 4 Department of Health Outcomes and Behavior, Moffitt Cancer Center, Tampa, FL 33612, USA.; 5 Department of Radiation Oncology, Sidney Kimmel Medical College and Cancer Center at Thomas Jefferson University, Philadelphia, PA 19107, USA.; 6 Centre for Health Services Research, The University of Queensland, QLD 4102, Australia.; 7 Applied Health Research Centre, Li Ka Shing Knowledge Institute, St Michael’s Hospital, Toronto M5B 1W8, Canada.; 8 Divisions of Rheumatology and Allergy and Clinical Immunology, Johns Hopkins University, Baltimore, MD 21224, USA.; 9 Johns Hopkins University Sidney Kimmel Comprehensive Cancer Center, Baltimore, MD 21231, USA.; 10 University of Michigan Rogel Cancer Center, Ann Arbor, MI 48109, USA.; 11 Department of Behavioral Science, Center for Health Equity Transformation and Markey Cancer Center, University of Kentucky College of Medicine, Lexington, KY 40536, USA.

**Keywords:** Immunotherapy, immune checkpoint modulators, quality of life, immune-related adverse events, patient reported outcomes, cancer, oncology

## Abstract

**Aim:**

To develop a comprehensive item library of patient-reported, immunotherapy-related adverse events (irAEs) that draws from and expands on the Functional Assessment of Chronic Illness Therapy (FACIT) Measurement System.

**Methods:**

Literature review and iterative expert input. Based on a literature review of irAEs, we developed a framework of immunotherapy classes and their associated symptoms. Clinical experts then reviewed iterations of symptom summaries and item maps linked to the immunotherapy framework. Experts provided content review and feedback was shared across experts until consensus was reached. The iterative process facilitated creation of a Primary Symptom List associated with immune checkpoint modulators (ICMs), drawn from the larger set of symptoms. Existing FACIT items were mapped to the symptom list, and new items were written as needed to create the item library.

**Results:**

The full item library of irAEs is comprised of 239 items, covering 142 unique symptoms across 75 inflammatory reactions/immune conditions. A subset of 66 items comprises a Primary Symptom List considered most common/relevant to ICM treatment. This includes gastrointestinal, skin, pulmonary, neurologic, musculoskeletal, and multiple miscellaneous and constitutional symptoms.

**Conclusion:**

The FACIT Immunotherapy Item Library is a compilation of 239 self-report items that capture the wide range of AEs experienced by people receiving immune treatments. A subset of 66 items comprises a Primary Symptom List meant for ICM therapy. Use of items selected from this library is encouraged in clinical research and clinical practice evaluation.

## INTRODUCTION

The emergence of immune checkpoint modulators (ICMs) in cancer treatment has produced both optimism and uncertainty among oncologists and patients. Over the last decade, the demonstrated efficacy of cytotoxic lymphocyte antigen-4 (CTLA-4) inhibitors, programmed cell death protein-1 (PD-1) inhibitors, and PD-1 ligand (PD-L1) inhibitors to induce prolonged responses in advanced cancers has been well-documented^[1–3]^. Such results were first noted in the treatment of metastatic melanoma, and the United States Food and Drug Administration (FDA) has since approved the use of ICMs for the treatment of non-small cell lung cancer, renal cell carcinoma, Hodgkin lymphoma, urothelial carcinoma, head and neck cancers, and other tumors^[4,5]^. A recent meta-analysis of randomized clinical trial data involving the use of anti-PD1/PD-L1 monoclonal antibodies to treat more than 6500 patients confirmed increased overall response rates when compared to usual care, including chemotherapy and targeted therapy^[6]^. While the greatest gains were seen in patients with melanoma and those treated in the first-line setting, improved response rates associated with ICMs were seen across all tumor types. However, despite justifiable optimism surrounding ICMs and other biologic therapies, their relative success is tempered by uncertainty as to which patients are likely to benefit, and new challenges in the detection and management of a host of potential short- and long-term adverse events unique to immune-mediated therapy^[7]^. Clinicians and patients discussing treatment options for advanced cancer must balance this ambiguity alongside their desire to pursue every possible path toward longer life^[8]^.

ICM treatment tolerability can complicate treatment decision-making, even for those patients who are promising candidates for ICM therapy. Immune-related adverse events (irAEs), including gastrointestinal, dermatologic, endocrinologic, cardiopulmonary, musculoskeletal, and other autoimmune complications, occur in many patients treated with ICMs^[4,9–15]^. One study found that adverse events occur in up to 90% of patients treated with CTLA-4 and 70% of patients treated with anti-PD-1/PD-L1 agents^[13]^. In trials using single anti-PD-1/PD-L1 agents, the rates of Grade 3 and 4 toxicities capable of hospitalizing patients and leading to treatment discontinuation ranged from 10% to 20%^[7]^. IrAEs are diverse, can impact almost any organ system, and can be potentially life-threatening (e.g., colitis and pneumonitis). They include the development of inflammatory and autoimmune conditions with unpredictable onset, as well as delayed and late effects that may persist long after treatment ends and require ongoing treatment with immunosuppressive agents in some cases^[5,16]^.

In the oncology community, recent efforts to catalog and classify ICM-associated toxicities and to set standardized management guidelines are helping treating clinicians understand the complexities of immunotherapy and to better diagnose and manage their patients’ symptoms. Importantly, between 2017 and 2018, extensive guidelines for the management of immunotherapy-related toxicities were issued by the National Comprehensive Cancer Network in collaboration with the American Society of Clinical Oncology, the European Society for Medical Oncology, and the Society for Immunotherapy of Cancer Toxicity Management Working Group^[4,9,10]^. Nevertheless, the era of immunotherapy is still in its infancy; more data are needed to understand risk factors, clinical phenotypes, and the nature, trajectory, and severity of short- and long-term immunotherapy-related toxicities, as well as the requirements for and responses to other immunosuppressive agents. These data are needed to more accurately inform clinicians and patients about how such toxicities affect the therapeutic risk-benefit ratio associated with ICMs. The availability of a patient-reported, targeted symptom assessment of irAEs for use in both the clinical trial and clinical practice settings would help address this important need.

### Patient-reported outcome assessment in immunotherapy

Patient-reported outcome (PRO) data represent a rich and currently underutilized resource that can contribute significantly to our knowledge of patients’ experiences with symptoms of cancer, its treatment, and impact on health-related quality of life (HRQOL). PROs are defined as “any report of the status of a patient’s (or person’s) health condition, health behavior, or experience with healthcare that comes directly from the patient, without interpretation of the patient’s response by a clinician or anyone else”^[17]^. In clinical trials, PROs enhance understanding of treatment toxicity, HRQOL, and treatment value^[18–22]^. In clinical practice, PROs aid early symptom detection, symptom management, and treatment decision making^[23–27]^.

PRO measurement systems frequently used in oncology include the Functional Assessment of Chronic Illness Therapy Measurement System (FACIT), the European Organisation for Research and Treatment of Cancer (EORTC), and the Patient-Reported Outcomes Measurement Information System (PROMIS) ^[28–30]^. FACIT, EORTC, and PROMIS systems provide valid and reliable measurement of disease-related physical, functional, social, and emotional concerns and some treatment toxicities. More recently, Basch and colleagues developed the Patient-Reported Outcomes-Common Terminology Criteria for Adverse Events (PRO-CTCAE™) measurement system, a compendium of PRO items uniquely targeted to the assessment of symptomatic treatment-related toxicities in oncology care^[31]^. PRO-CTCAE allows for the use of individually selected questions drawn from a pool of over 100 items organized into 14 National Comprehensive Cancer Network (NCCN)-designated toxicity domains, advancing the acceptability of customizable forms. Although these measurement systems have advanced our understanding of treatment toxicity and HRQOL, they were developed prior to the widespread use of ICMs. To date, there are no established PRO measures that assess the full range of common and unique symptoms of AEs related to ICM treatment^[32,33]^.

We therefore set out to develop an inclusive list and a library of patient-reported items that assess adverse events associated with immunotherapy, intended to be conceptually and structurally similar to the PRO-CTCAE, enabling selective assessment of subsets for specific use. In this paper, we report on the development of a conceptual framework and extensive FACIT Immunotherapy Item Library. We also describe the parallel development of a Primary Symptom List, a representative subset of library items selected by our expert panel via a modified Delphi technique. The FACIT Immunotherapy Item Library offers targeted assessment options, enables custom form creation, and has the flexibility needed to accommodate new content in order to stay current with the changing landscape of immunotherapy treatment.

## METHODS

**Figure 1.**
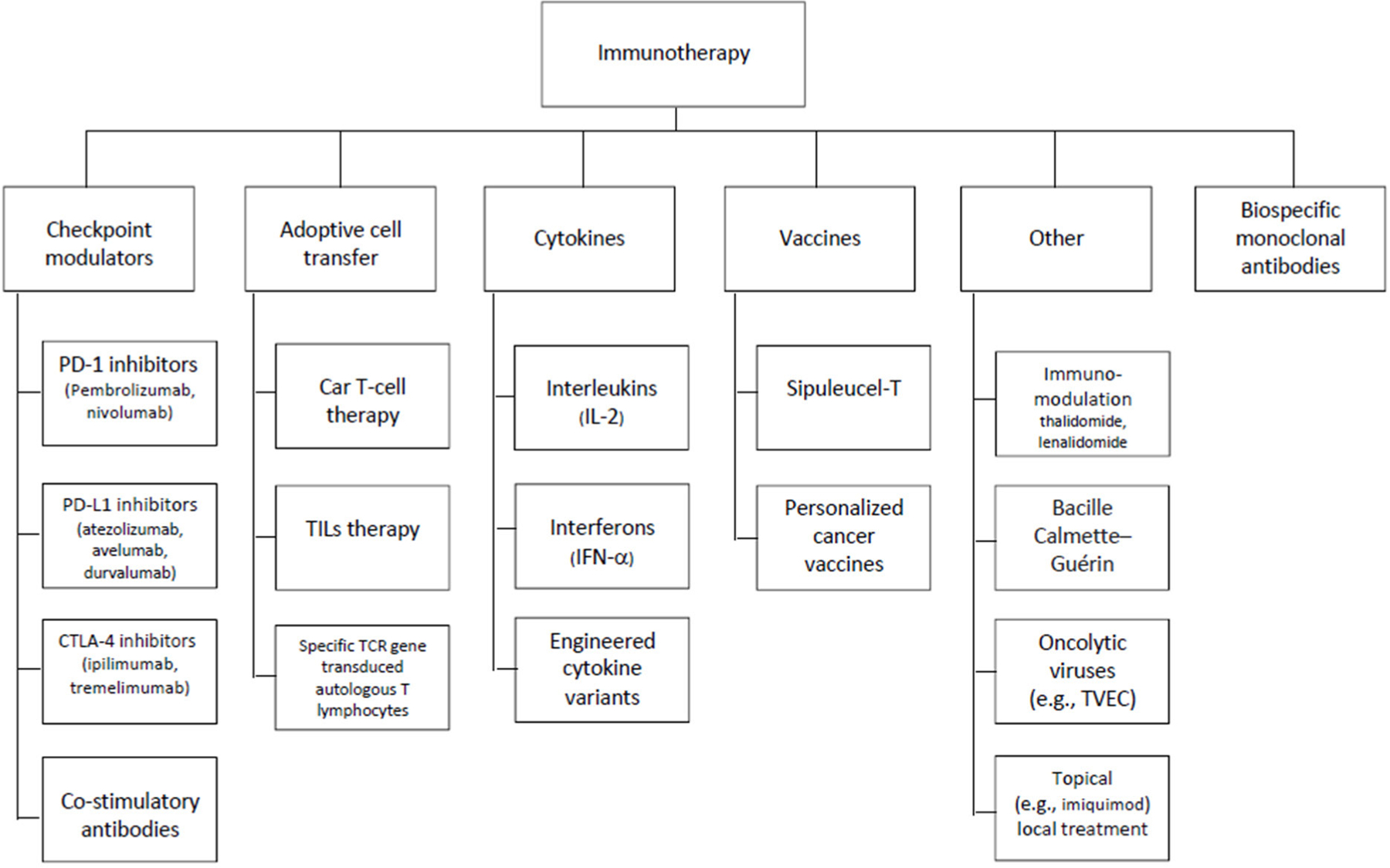
Classes of immunotherapy

A consortium of investigators across several institutions, represented by the author list, convened around a commitment to produce a set of PRO items to assess symptoms associated with immunotherapy. Figure 1 depicts the range of immunotherapies we considered. The array of treatments covered by Figure 1 produced a large number of candidate adverse events to capture, leading us to recognize that a modular approach to assessment would be most practical, perhaps even necessary, for most applications.

**Figure 2.**
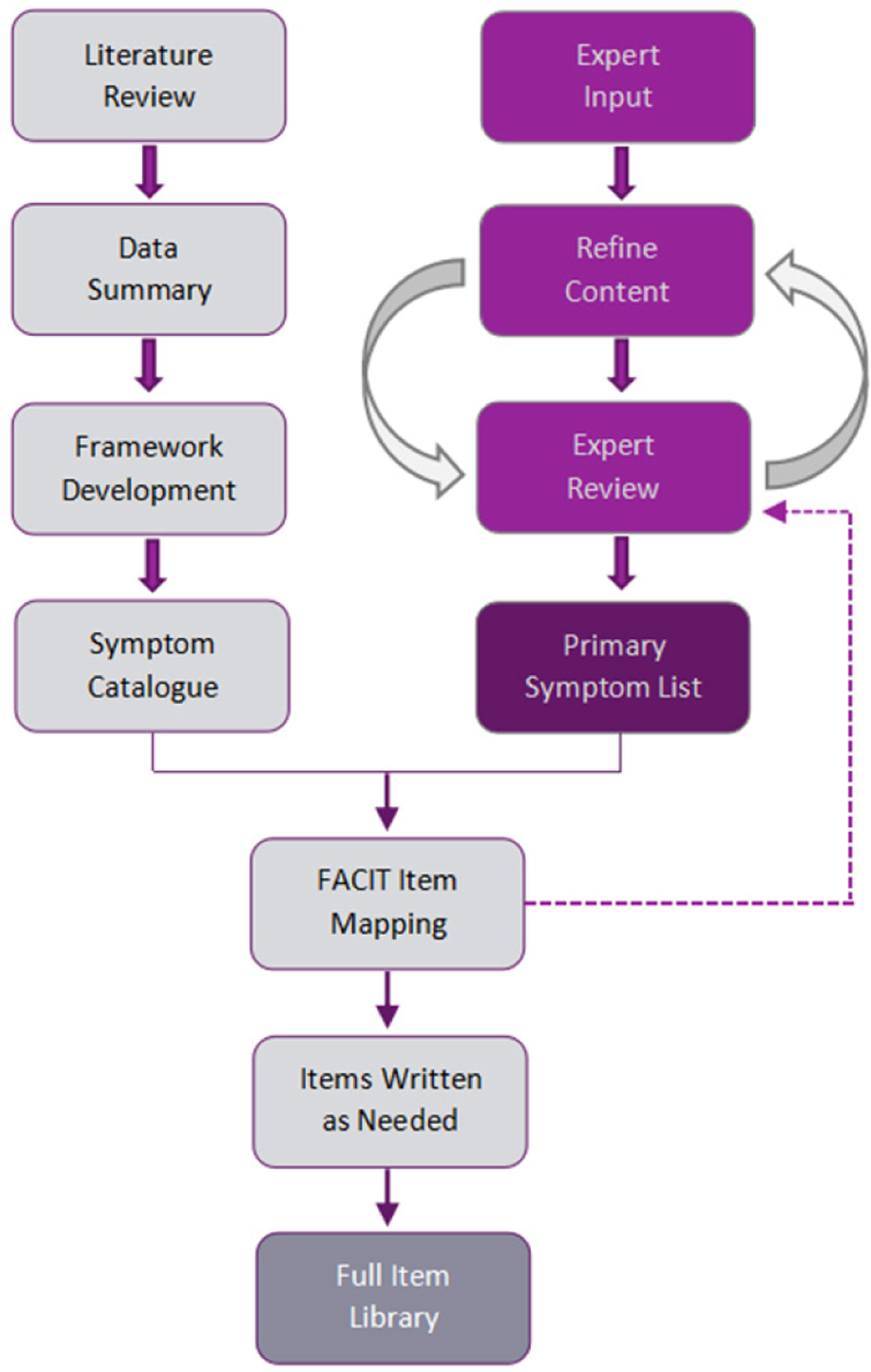
Development process. FACIT: Functional Assessment of Chronic Illness Therapy

Although our ultimate goal is to catalogue immunotherapy toxicities generally (and as depicted in Figure 1), we began with where the most experience and interest lies, namely the adverse effects associated with immune checkpoint modulators (ICMs; Figure 1, Column 1). We therefore expected this would be an exercise in identifying common toxicity profiles and their associated symptoms, followed by a prioritizing of item content. We reviewed published literature to inform an immunotherapy construct definition and conceptual framework. We mapped identified symptom experiences and impact onto previously developed survey items from the FACIT Measurement System, modifying existing items and drafting new items as necessary. This process produced a working version of an irAE item library. A shorter list of the more common and clinically relevant symptoms was developed based on iterations of symptom review and feedback from an expert panel, and then incorporated into the library. Figure 2 depicts our development process, representing parallel tracks of literature review and expert input.

### Expert input

A convenience sample of clinicians and researchers whose expertise aligned with project goals was identified to provide ongoing input and guidance during the ICM Primary Symptom List development. Experts with clinical experience treating patients with ICM therapies or researching ICM-related AEs were included in an advisory panel. We used panel input to compile an initial list of commonly seen and/or clinically relevant ICM-related adverse event symptoms. Then, we used iterations of symptom summaries, item mapping, content review, and feedback (informally through e-mail and formally via online surveys) for consensus building and to facilitate selection of final items for the Primary Symptom List. We used a modified Delphi technique for formal online surveying. The modified Delphi technique allows for consensus building through a sequence of two or more rounds of panel member review and feedback until sufficient agreement has been obtained regarding priority content^[34,35]^.

### Literature review

The goals of the literature review were: (1) to select recently issued guidelines for the identification and management of irAEs to help guide the development of a conceptual framework; (2) to identify ICM-related toxicity profiles and their associated organ-system based conditions; and (3) to catalogue and summarize key patient symptoms from the current literature on ICM therapies. As our goal was to capture a representative range of information to inform the conceptual framework and document treatment-related toxicities, we conducted a targeted literature review. Our main data sources were PubMed, Embase, and CINAHL. The search strategy utilized keyword terms associated with ICMs, cancer, and toxicities combined with Boolean operators (“OR” and “AND”) to identify immunotherapy keywords in the titles and abstracts of articles. We also searched select textbooks and reputable websites (e.g., those of the American Society of Clinical Oncology, the National Comprehensive Cancer Network, and the European Society for Medical Oncology) for articles or chapters relating to ICM side effects and their clinical management. Inclusion criteria for relevant articles or chapters included those written in English and published in peer-reviewed journals within the last five years or in similarly recent authoritative medical textbooks. Articles and chapters that fit the inclusion criteria were assigned to a team member for full text review, and relevant content was summarized in an Excel spreadsheet.

### Framework development

For framework guidance, we reviewed publications related to recently issued guidelines for the identification and management of irAEs. We selected seminal publications, highlighted key thematic information from each, and organized our framework accordingly. Collectively, this provided a conceptual framing for PRO measurement in the context of a complex matrix of ICM-related adverse events.

### Data summaries

From our literature review, we extracted all relevant information regarding ICM-induced inflammatory reactions and immune conditions, as well as their associated toxicity profiles and symptomatology. Data were summarized in Excel spreadsheets, which allowed for detailed descriptions of symptom experience. We added expert-identified symptoms to the composite and highlighted these for Primary Symptom List consideration. Symptoms identified for possible inclusion in the library were reviewed by a team of experts with extensive experience in PRO measure development for relevance, redundancy, and appropriateness as measured by self-report. Symptoms for the Primary Symptom List were iteratively reviewed by our expert panel. Redundant or similar symptoms were collapsed, and symptoms that could not be measured via patient-report were excluded.

### Item mapping

Once a comprehensive list of symptomatology had been finalized, we organized immune conditions (inflammatory responses and autoimmune syndromes) and symptom profiles according to our conceptual framework. We then mapped symptoms onto existing items in the FACIT Measurement System via the FACIT Item library (https://wizard.facit.org), filling gaps by modifying existing items when possible and writing new items as necessary^[28,36]^. The FACIT Measurement System includes over 100 distinct self-report questionnaires that assess a wide variety of disease- and treatment-related symptoms, functional abilities (physical, mental, and social), general perceptions of health and well-being, and other aspects of health-related quality of life. The FACIT item library is a collection of more than 700 unique health-related PRO questions that appear in the FACIT Measurement System. Most FACIT items have demonstrated face and content validity, and were created with direct input from patients and expert clinicians. Many items have also been translated into up to 70 languages using a standardized, rigorous translation methodology^[37,38]^ and tested for comprehension by native speakers.

For the immunotherapy library, our team identified the most suitable item for symptom assessment, and then included other items related to impact or bother as appropriate. For the Primary Symptom List, when more than one FACIT item was available, our expert panel assisted in selecting the top 1–3 items targeted to symptom assessment in the setting of ICM therapy.

## RESULTS

### Expert input

Our panel of experts was tasked with identifying a Primary Symptom List of ICM-related adverse events. Experts were MDs (*n* = 7), PhDs (*n* = 4), and a PhD candidate (*n* = 1), with 2–8 years of experience treating cancer patients with ICM therapies and/or researching the incidence, nature, and HRQOL impact of irAEs in the context of collaborative clinical and translational trials.

Experts identified an initial list of 61 commonly seen or clinically relevant irAE symptoms for ICM Primary Symptom List inclusion. We then mapped symptoms onto existing items in the FACIT item bank, which included those that address symptom presence, symptom bother and/or emotional or functional impairment associated with symptom experience (e.g., “I have stiffness or tightness in my joints” and “Joint stiffness limits my usual activities”). Mapped irAE symptoms yielded 153 unique FACIT items. For symptoms where there was no corresponding FACIT item, we adapted a similar, existing item (e.g., replaced “eyes” with “mouth” for item HN3 “My mouth is dry”) or wrote a new item [e.g., “I am bothered by vitiligo (white patches on my skin)”].

We then asked our expert panel to review selected PRO items, and identify the most relevant ones for ICM treatment assessment. We used consensus and input from our internal team of measure developers to refine the list to 47 items covering 32 symptoms, and then sent it back to the panel members for a second iteration of review. During this review our panel helped: (1) identify items that were a misfit or symptoms still missing from the list; (2) confirm that item text targeted desired symptom assessment; and (3) refine item wording for newly written items as needed. Again, we used consensus and internal team input to hone the list to 53 primary and 13 supplemental items, and then sent it back to our panel for one final review to confirm content and identify any lingering item(s) that should be omitted or symptom(s) that should be included.

The final Primary Symptom List includes 66 items assessing 48 symptoms deemed common or clinically relevant to ICM adverse event symptom monitoring. Symptoms include: gastrointestinal (diarrhea, constipation, reflux/heartburn, abdominal pain, abdominal swelling, abdominal bloating, nausea, vomiting, blood in stool, and mouth sores); skin (rash, itch, dryness, cracking/peeling, blistering, vitiligo, sensitivity to sun, and pain); lung (shortness of breath, cough, wheezing, and chest pain); neurologic (coordination and balance); musculoskeletal (joint pain, joint stiffness, swelling, muscle weakness, and muscle pain); and miscellaneous/constitutional (fatigue, headache, weight gain, weight loss, appetite, lightheaded/dizziness, blurry vision, urinary frequency, fevers, chills, nervousness, palpitations, sweating, dry mouth, dry eyes, sandy/gritty eyes, sinus pain, incidence of new allergies, and acute treatment reactions). Of the 66 items, 53 are primary and 13 supplemental [Table 1]. All symptoms are part of the larger FACIT Immunotherapy Item Library.

### Conceptual framework development

**Figure 3.**
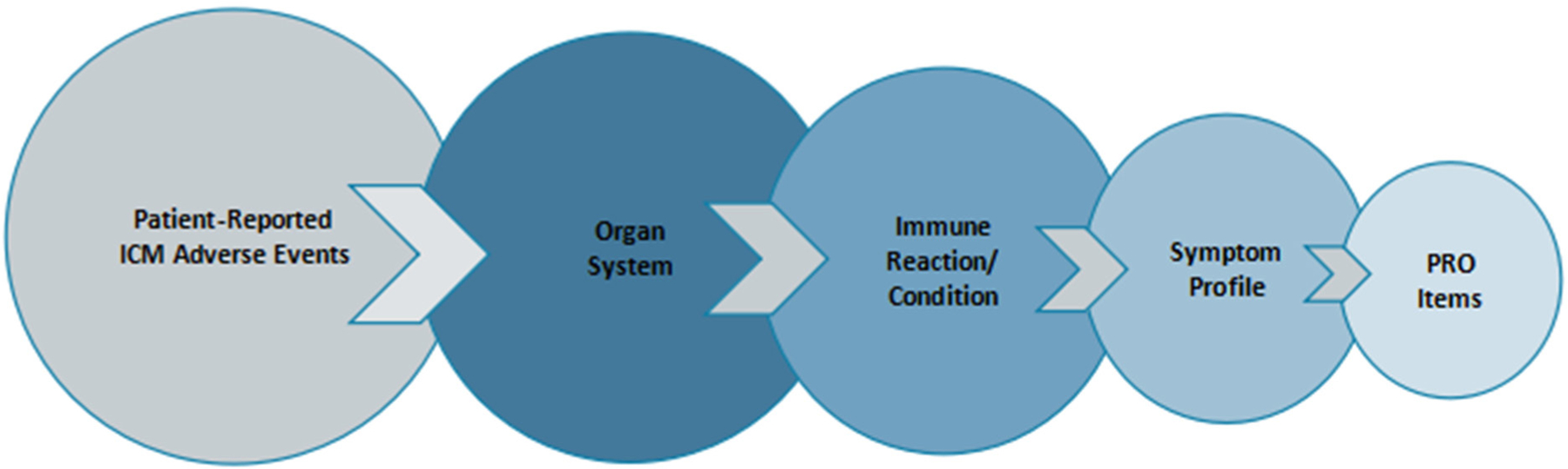
Conceptual framework. PRO: patient-reported outcome; ICM: immune checkpoint modulators

To inform our conceptual framework, we used recently issued guidelines for the identification and management of immunotherapy-related toxicities from The American Society of Clinical Oncology, in collaboration with the National Comprehensive Cancer Network, the European Society for Medical Oncology, and the Society for Immunotherapy of Cancer Toxicity Management Working Group^[9,10,39]^. Collectively, these guidelines identify primary ICM-related immune conditions by organ system, highlighting key symptoms for each, which we then used as the organizing conceptual structure of our immunotherapy library. The content of the item library includes a taxonomy of patient-relevant endpoints according to: organ system > immune reaction/condition > symptom profile > PRO questions [Figure 3].

### Literature review

Our literature review yielded 25 articles documenting irAE incidence, management guidelines, toxicity profiles, and associated symptomatology^[4,9,11–15,40–57]^. From these, we identified 75 possible inflammatory reactions/immune conditions across 11 organ systems, ranging from very common to rare. Organ systems were categorized as: (1) cutaneous; (2) gastrointestinal; (3) hepatic; (4) lung; (5) endocrine; (6) musculoskeletal; (7) renal; (8) nervous; (9) hematologic; (10) cardiovascular; and (11) ocular. Identified inflammatory reactions/conditions and their symptom profiles were summarized, and all associated symptoms measurable by patient report were retained for PRO item mapping or development [Table 2]. We identified a total of 142 unique symptoms, several of which overlap immune conditions. Symptoms include: gastrointestinal [abdominal bloating, abdominal fullness, abdominal pain, abdominal swelling/distention, pain radiating to the back, blood in stool, diarrhea, frequent bowel movements, constipation, heartburn (burning chest pain), regurgitation, nausea, vomiting, vomiting blood, blood in stool, rectal bleeding, and mouth sores]; skin (rash, rash-itch, rash-blisters, rash-pain, rash-burning, skin depigmentation, skin itch, skin dryness, skin cracking/peeling, skin blisters, skin pain, skin thickening, skin thinning, nail pitting, nail ridging, touch sensitivity, sun sensitivity, hair loss, and face reddening); lung (shortness of breath, shortness of breath lying down, cough, cough lying down, wheezing, chest pain, chest pressure, and edema); neurologic (coordination, balance, memory, confusion, disorientation, eyelid droop, facial droop, face pain, stiff neck, numbness, tingling, burning pain, back pain, paralysis, sweating-decreased ability, muscle-weakness, sleepiness, drooling, double vision, light sensitivity, jaw pain, voice changes, swallowing difficulty/choking, bladder control, and bowel control); musculoskeletal (joint pain, joint stiffness, joint swelling, muscle pain/soreness, decreased range of motion, functional interference, and bone pain); eyes (eye pain, eye itch, eye redness, dry eyes, sandy/gritty eyes, crusty eyes, watery eyes, pain with eye movement, eyelid swelling, and eyelid itch); hematologic (bleeding, bleeding easily, bleeding gums, bleeding nose, pinpoint bleeding-petechiae, bruising, bruising easily, and lymph swelling); mood (depressed, nervous/anxious, irritable, and mood changes); and miscellaneous/constitutional (general pain, fatigue, headache, weight loss, weight gain, appetite increase, appetite decrease, lightheaded/dizziness, malaise, weakness, paleness, jaundice, fevers, low-grade fever, chills, sweating excessive/nocturnal, palpitations, dry mouth, taste changes, thirst, sinus pain, blurred vision, decrease vision, loss of vision, vision floaters, impaired color vision, pain radiating to the back, swelling, urinary frequency, urinary infrequency, foamy urine, blood in urine, dark urine, tooth decay, moon face appearance, heat intolerance, cold intolerance, food sensitivity, cold fingers, cold toes, groin pain, new allergies, and acute treatment reactions).

### Item mapping

For the FACIT Immunotherapy Item Library, 142 unique symptoms were identified for item mapping, which included 48 from the Primary Symptom List. Once symptom content had been fully determined, the study team met to align symptoms with candidate items from the overall FACIT item library. We identified all items related to a given symptom (e.g., for fatigue, there were 16 items covering fatigue experience and impact), and then selected the one(s) that best captured symptom experience or impact related to ICM therapy. We used descriptions from experts and the literature to corroborate item selection. This process yielded a final total of 239 PRO items retained for immunotherapy item library inclusion, which includes a subset of 66 items from the Primary Symptom List. The full set of immunotherapy items can be found at https://wizard.facit.org/.

## DISCUSSION

The FACIT Immunotherapy Item Library was developed with input from the literature, clinicians, and researchers and constitutes the first published compendium of PRO items targeting the vast array of irAEs in ICM therapy. Library content is aligned with current practice guidelines and conceptually organized by organ system, inflammatory reaction/condition, and their associated toxicity symptom profiles. PRO items address adverse events associated with immunotherapy generally, and a subset of items for ICM treatments, which include the related functional, social, and/or emotional impact of those symptoms when applicable. Use of items from the widely-used FACIT Measurement System ensures PRO assessment based on well-validated items that have undergone extensive face and content validity, are written at the sixth grade reading level or less, and are available in as many as 70 languages. All items use a standard five-point Likert scale (0 = not at all; 1 = a little bit; 2 = somewhat; 3 = quite a bit; and 4 = very much) and seven-day recall period.

**Figure 4.**
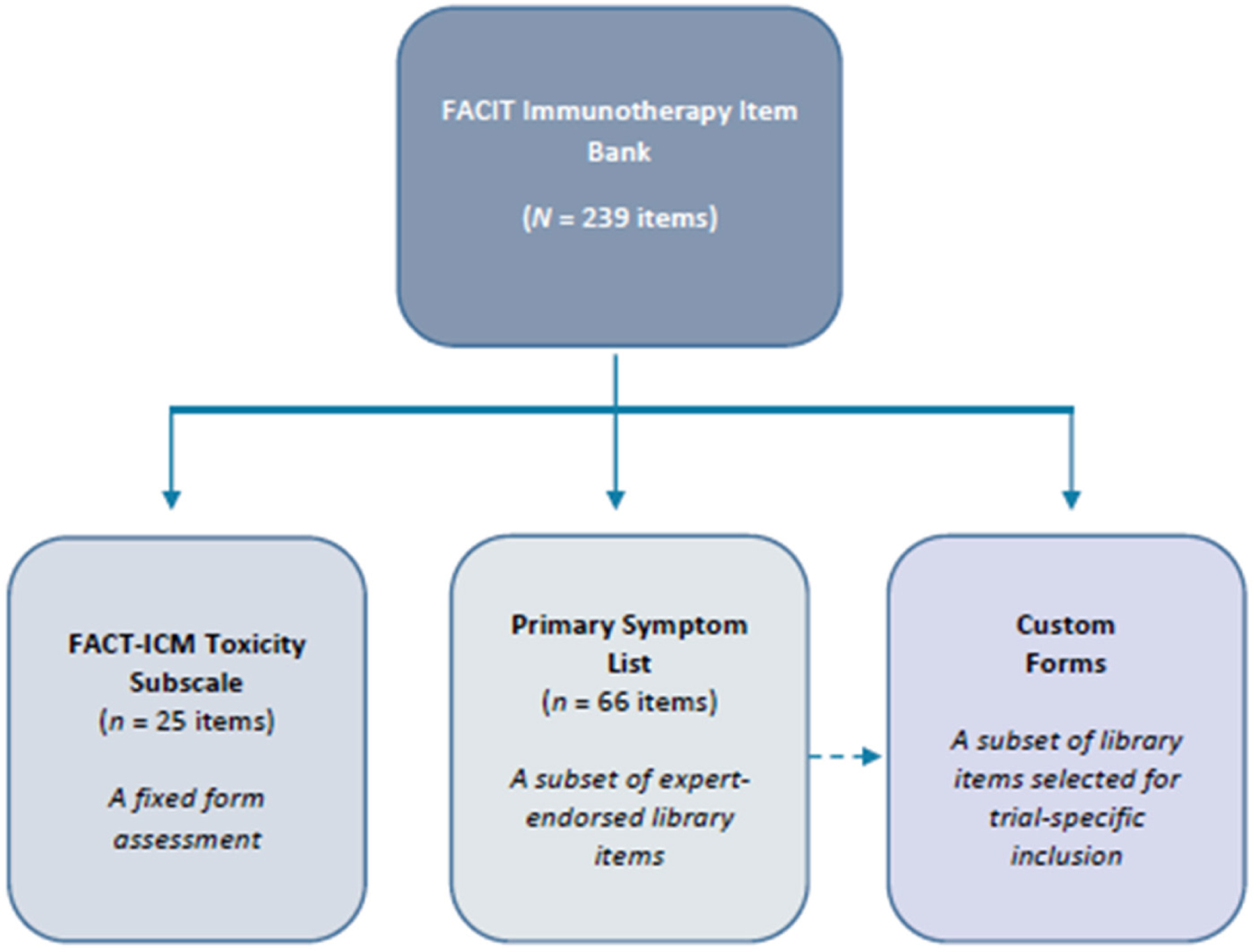
Current FACIT immunotherapy assessment options. FACIT: functional assessment of chronic therapy

Library format permits for custom form development, as well as expansion based on new or combination therapies and into other immunotherapy classifications. Asking every question in the library would of course be burdensome to a patient in clinical research, especially when other questionnaires are also likely to be desired to measure disease-related symptoms, function, health perceptions, and quality of life. We advocate for the thoughtful, judicious use of subsets of these questions, assembled by a survey builder (FACIT Build-a-PRO; https://wizard.facit.org/), to track the most likely or relevant adverse events in a given trial. This practice of custom building has been recommended for use of the PRO-CTCAE^[31]^ and we endorse that same approach here. Users can draw items from the full library (*n* = 239) or select from a targeted subset of expert-endorsed items (Primary Symptom List; *n* = 66). The Functional Assessment of Cancer Therapy-Immune Checkpoint Modulator (FACT-ICM) is also newly available from Hansen and colleagues - a 25-item toxicity subscale for patients receiving ICM therapy^[58]^. The FACT-ICM was developed using a mixed methods approach and is designed to assess HRQOL in the context of ICM irAEs, providing value in research and clinical settings related to symptom assessment and quality of life. Hansen and colleagues offer users a fixed-form assessment, developed in accordance with FDA PRO guidelines, with items that are included in the overall item library^[17,58]^. A second initiative by Jim and colleagues aims to develop a FACT measure with items reflective of common patient-reported toxicities in lung cancer patients receiving immunotherapy^[59]^. They have analyzed PRO and other data from the Addario Lung Cancer Foundation international patient registry to describe patient-reported toxicities and quality of life outside the context of a clinical trial in lung cancer patients treated with immune checkpoint inhibitors. Figure 4 depicts current FACIT immunotherapy adverse event assessment options.

In conclusion, using literature review, expert consensus, and previously developed items from the FACIT Measurement System, we developed an item library that collectively targets full-spectrum PRO assessment of ICM-related adverse events. Library item selection for tailored forms allows for targeted, concise, clinically relevant measurement of symptoms and concerns to monitor when assessing the value of ICM treatment for patients with cancer. Collection of PRO data in the emerging field of immuno-oncology will make it possible to more accurately assess the added adverse event burden associated with new combination regimens and support longitudinal studies to evaluate the long-term effects of both single-agent and combination therapies. At the point of care, PRO data can support shared decision-making between clinicians and patients, and facilitate early detection of, and communication among, the wide range of specialists required to provide comprehensive care for immunotherapy-related adverse events. Direct patient report of immunotherapy-related adverse events may help to identify early symptoms before clinical signs are manifest to the clinician. For example, patients report joint pain and stiffness prior to clinical recognition of myalgia/arthralgia. Earlier detection and recognition of these adverse effects may enable early symptom management and/or treatment modifications that contribute to better quality of life over time.

## Figures and Tables

**Table 1.** Primary symptom list

I have diarrhea (diarrhoea)	I have pain in my chest
I have to limit my activities because of diarrhea (diarrhoea)*	I feel lightheaded (dizzy)
I must move my bowels frequently to avoid accidents*	My eyesight is blurry
I am constipated	I have trouble with coordination
I am bothered by reflux or heartburn	I am able to maintain my balance*
I have pain in my stomach area	I have pain in my joints
Stomach pain interferes with my daily functioning*	I have stiffness or tightness in my joints
I have swelling in my stomach area	Joint stiffness or tightness limits my usual activities
I feel bloated*	Joint pain limits my usual activities
I have nausea	I have weakness in my arms or legs
I have been vomiting	I am bothered by muscle pains
I have noticed blood in my stool	I am bothered by swelling in certain areas of my body
I have a loss of appetite	I get headaches
I feel fatigued	I am bothered by headaches*
My fatigue keeps me from doing the things I want to do*	I urinate more frequently than usual
I have a lack of energy	I am losing weight
I feel tired*	I am bothered by a change in weight*
My skin (or scalp) itches	I have had fevers
My skin (or scalp) is dry or “flaky”	I am bothered by fevers (episodes of high body temperature)*
I am bothered by dry skin	I have had chills
I am bothered by cracking or peeling of my skin	I am bothered by chills*
I am bothered by blistering of my skin	I feel nervous
I am bothered by vitiligo (white patches on my skin)	I have episodes of heart racing
The skin on my feet hurts	I am bothered by sweating
Pain on the bottom of my feet interferes with my walking	My eyes are dry
I have mouth sores	My eyes feel sandy or gritty
I am bothered by a skin rash	My mouth is dry
The skin on my hands hurts	My mouth and throat are dry*
I am bothered by a change in my skin’s sensitivity to the sun	I am bothered by dry mouth*
I have pain in my hands or feet when I am exposed to cold temperatures	I have pain in my sinus area
I have been short of breath	I am bothered by side effects of treatment
I have been coughing	I am bothered by new allergy-like reactions (e.g., to foods, insects, pollen)
I have been wheezing (whistling sound when I breathe)	I am bothered by short-term treatment reactions that I experience immediately after, or within 24 h of, an infusion (such as chills, dizziness, hives, rashes lasting no more than 24 h)

* Designates a supplemental item

**Table 2.** ICM-related inflammatory reactions/autoimmune conditions and their associated symptoms

Organ system (*n* = 11)	Inflammatory reaction/condition (*n* = 75)	Symptoms (*n* = 142 unique symptoms)
Cutaneous	Rash; pruritus; psoriasis; pitted or ridged nails; vitiligo; Steven’s Johnson’s syndrome; Lyell’s syndrome (toxic epidermal necrosis); alopecia; Sweet syndrome	Rash, rash-pain, rash-blister, rash-itch, skin-itch, skin-dry/cracked, skin-peeling, skin-burning, skin-thickened, nails-pitted, nails-ridged, skin-depigmentation, fever, skin-pain, mouth-blisters, mouth-pain, malaise, hair loss
Gastrointestinal	Colitis; pancreatitis; stomatitis/oral mucositis; lichenoid mucositis; esophagitis; gastritis; celiac disease; reflux; cholangitis; intestinal perforation; cholecystitis; gastric hemorrhage; paralytic bowel obstruction	Diarrhea, abdominal-pain, weight-loss, fever, nausea, vomiting, blood in stool, fatigue, pain radiating to the back, shortness of breath, mouth-sores, mouth-pain, taste changes, swallowing-difficulty, swallowing-pain, chest-pain (burning), heartburn, abdominal-fullness, appetite-loss, abdominal-bloating, regurgitation, skin-itchy, eyes-itchy, mouth-dry, urinary-infrequency, thirst-increased, abdominal-distention, sensitivity-food, vomiting-blood, rectal bleeding, constipation
Hepatic	Hepatitis; acute liver failure	Fatigue, abdominal-pain, joint-pain, jaundice, abdominal-swelling, nausea, vomiting, malaise, disorientation, confusion, sleepiness, dark urine
Renal	Interstitial nephritis; nephrotic syndrome; acute kidney injury	Weight-gain, edema, fever, urine-blood, nausea, vomiting, urinary-frequency, urinary-infrequency, urine-foamy, appetite-loss, shortness of breath, fatigue, confusion, weakness
Lung	Pneumonitis; pleuritis; sarcoidosis/sarcoid-like granulomatosis	Shortness of breath, cough, fatigue, appetite-loss, weight-loss, chest-pain, fever, joint-pain, joint-swelling, lymph swelling, rash, vision-blurred, eyes-pain
Endocrine	Hypophysitis/hypopituitarism; primary hyperthyroidism; primary adrenal insufficiency; hyperglycemia/autoimmune diabetes; diabetic ketoacidosis; primary hypoparathyroidism; thyrotoxicosis/hyperthyroidism; Cushing’s syndrome	Headache, fatigue, muscle-weakness, nausea, appetite-loss, weight-loss, vision-blurred, intolerance-cold, intolerance-heat, constipation, skin-dry, weight-gain, muscle-pain/ache, joint-pain, joint-stiffness, joint-swelling, depression, memory impairment, abdominal-pain, nausea, vomiting, diarrhea, lightheadedness/dizziness, thirst-increased, urinary-frequency, appetite-increase, irritability, anxiety, palpitations, bone pain, sweating-excessive, bowel-frequent movements, bruising-easy, face-reddening, skin-thinning, moon face appearance, mood changes
Musculoskeletal	Myalgia; arthralgia; inflammatory arthritis; dermatomyositis; myopathy; inflammatory myositis; polymyalgia rheumatica; Sicca-Sjogren’s like syndrome; sarcoidosis-like reactions	Muscle-pain/ache, joint-pain, joint-stiffness, joint-swelling, joint-decreased range of motion, skin- swelling, back pain, skin-rash (violet or red), skin-itch, skin-pain, sensitivity-touch, muscle-weakness, muscle-soreness, fatigue, swallowing-difficulty, shortness of breath, joint-ache, groin-pain, fever (low-grade), malaise, depression, appetite-loss, weight-loss, mouth-dry, face-pain, tooth-decay, mouth-pain, eyes-dry, taste changes, shortness of breath, lymph swelling, cough, wheezing, eyes-pain, eyes-redness, functional interference/eat and sleep interference
Nervous	Neuropathy; Guillain-Barre syndrome; myelopathy; transverse myelitis; meningitis; encephalitis; myasthenic syndrome/myasthenia gravis; facial nerve palsy	Numbness, pain, pain-burning (nerve), muscle-weakness, coordination-difficulty, paralysis, intolerance-heat, sweating-excessive, sweating-decreased ability, dizziness/lightheadedness, tingling, numbness, balance-problems, urine-loss of control, bowel-loss of control, sensitivity-touch, fever, headache, stiff neck, nausea, vomiting, confusion, sleepiness, sensitivity-light, muscle-pain/ache, joint-ache, fatigue, eyelid-drooping, muscle-weakness (that is better with rest), vision-double, swallowing-difficulty, voice changes, drooling, facial droop, jaw pain, taste changes
Hematologic	Hemolytic anemia; aplastic anemia; idiopathic thrombocytopenia purpura; pancytopenia; hemophilia	Fever, weakness, dizziness, confusion, urine-blood, paleness, jaundice, palpitations, bruising-easy, bleeding-pinpoint (petechiae), bleeding-easy, bleeding-gum, bleeding-nose, blood in stool, fatigue, shortness of breath, joint-pain, joint-swelling, lightheadedness/dizziness
Cardiovascular	Myocarditis; vasculitis/temporal arteritis; interstitial lung disease; pericarditis; cardiomyopathy; heart failure; pericardial effusion; Raynaud’s phenomenon	Chest pain, fatigue, shortness of breath, edema, palpitations, fever, headache, weight-loss, muscle-pain/ache, muscle-pain, sweating-nocturnal, rash, numbness, weakness, tingling, cough-dry, shortness of breath-lying down. cough-lying down, abdominal-swelling, chest-pressure, lightheadedness/dizziness, wheezing, weight-gain, urinary frequency-nocturnal, chest-fullness, fingers-cold, toes-cold, functional impairment such as reduced exercise intolerance
Ocular	Uveitis/choroiditis; conjunctivitis; scleritis; episcleritis; blepharitis; retinitis; orbital myositis	Eyes-redness, eyes-pain, sensitivity-light, vision-blurred, vision-floaters, vision-decreased, eyes-itchy, eyes-gritty, eyes-crusty, eyes-watery, eyes-pain with eye movement, eyelids-itchy, eyelids-red/swollen, vision-loss, vision-impaired color vision, vision-double, eyelid-drooping

ICM: immune checkpoint modulators
